# OX40L/OX40 Signal Promotes IL-9 Production by Mucosal MAIT Cells During *Helicobacter pylori* Infection

**DOI:** 10.3389/fimmu.2021.626017

**Published:** 2021-03-11

**Authors:** Siqi Ming, Mei Zhang, Zibin Liang, Chunna Li, Jianzhong He, Peiyu Chen, Shunxian Zhang, Xiaoli Niu, Shimei Deng, Lanlan Geng, Guoliang Zhang, Sitang Gong, Yongjian Wu

**Affiliations:** ^1^Department of Gastroenterology, Guangzhou Institute of Pediatrics, Guangzhou Women and Children's Medical Center, The Sixth Affiliated Hospital of Guangzhou Medical University, Qingyuan People's Hospital, Guangzhou, China; ^2^Center for Infection and Immunity, The Fifth Affiliated Hospital, Zhongshan School of Medicine, Sun Yat-sen University, Guangzhou, China; ^3^National Clinical Research Center for Infectious Diseases, The Third People's Hospital of Shenzhen, The Second Affiliated Hospital of Southern University of Science and Technology, Shenzhen, China; ^4^Department of Thoracic Oncology, The Cancer Center of The Fifth Affiliated Hospital of Sun Yat-sen University, Zhuhai, China; ^5^Department of Infectious Diseases, The Fifth Affiliated Hospital of Sun Yat-sen University, Zhuhai, China; ^6^Department of Pathology, The Fifth Affiliated Hospital of Sun Yat-sen University, Zhuhai, China

**Keywords:** MAIT cells, *H. pylori*, gastritis, IL-9, inflammation, OX40

## Abstract

Mucosal associated invariant T (MAIT) cells play a critical role in *Helicobacter pylori* (*H. pylori*)-induced gastritis by promoting mucosal inflammation and aggravating mucosal injuries (1, 2). However, the underlying mechanism and key molecules involved are still uncertain. Here we identified OX40, a co-stimulatory molecule mainly expressed on T cells, as a critical regulator to promote proliferation and IL-9 production by MAIT cells and facilitate mucosal inflammation in *H. pylori*-positive gastritis patients. Serum examination revealed an increased level of IL-9 in gastritis patients. Meanwhile, OX40 expression was increased in mucosal MAIT cells, and its ligand OX40L was also up-regulated in mucosal dendritic cells (DCs) of gastritis patients, compared with healthy controls. Further results demonstrated that activation of the OX40/OX40L pathway promoted IL-9 production by MAIT cells, and MAIT cells displayed a highly-activated phenotype after the cross-linking of OX40 and OX40L. Moreover, the level of IL-9 produced by MAIT cells was positively correlated with inflammatory indexes in the gastric mucosa, suggesting the potential role of IL-9-producing MAIT cells in mucosal inflammation. Taken together, we elucidated that OX40/OX40L axis promoted mucosal MAIT cell proliferation and IL-9 production in *H. pylori*-induced gastritis, which may provide potential targeting strategies for gastritis treatment.

## Introduction

Mucosal-associated invariant T (MAIT) cells are a class of innate-like T cells that recognize the small-molecule derivatives produced by microbes during riboflavin synthesis (1, 3, 4). Phenotypically, MAIT cells express a semi-invariant T cell receptor (TCR) (Vα7.2 in humans), recognizing specific antigens presented by major histocompatibility complex (MHC)-related protein-1 (MR1) with the monomorphic restriction, which is distinct with conventional T cells that recognize highly variable antigens *via* polymorphic MHC molecules (5, 6).

MAIT cells exert a fundamental function in host immune responses by responding quickly to invading pathogens without the need for clone expansion (1). Simultaneously, MAIT cells have an intrinsic effector-memory phenotype, capable of secreting pro-inflammatory cytokines rapidly, including TNF, IL-17, IFNα, IL-12, IFNγ, etc., to eliminate the microbes (6–8), thus playing critical roles in inflammation and infectious diseases. Changed frequencies of MAIT cell in peripheral blood and mucosal tissues are observed in a variety of inflammatory diseases, including autoimmune diseases (9), type1 diabetes (T1D) (10), type 2 diabetes (T2D) (11), rheumatoid arthritis (12), and gastritis (2). In recent years, MAIT cells have been suggested to participate in the immune responses against microbes in the human alimentary tract (13, 14). In inflammatory bowel diseases (IBD), a decreased frequency of MAIT cells in peripheral blood and an increased number in intestinal tissue were observed (15, 16), and the production of IL-17 and IL-22 by MAIT cells was increased (17, 18). Meanwhile, the existence of MAIT cells has been found in gastric mucosa, and the roles of MAIT cells are investigated. MAIT cells are observed to localize in proximity to *H. pylori* in the human gastric mucosa (2). Upon the recognition of *H. pylori* infected macrophage, MAIT cells can produce cytokines and exhibit cytotoxic activity (19). Otherwise, MAIT cells are associated with accelerated *H. pylori* gastritis in mice (2). However, the function of MAIT cells and regulatory factors in *H. pylori* gastritis are not fully clarified.

Gastritis induced by *H. pylori* infection is characterized by excessive mucosal inflammation, which is represented by the hypersecretion of mucus and cytokines, and inflammatory cell infiltration (20, 21). Gastritis may lead to gastric perforation, gastrorrhagia, ulcers, and even worse, stomach cancer after further development (22, 23). IL-9 is an emerging cytokine potentially involved in inflammatory diseases, especially IBD (24, 25). Induction of IL-9 is correlated with the severity of gut pathology, and blockage of IL-9 with neutral antibody suppresses the progression of colitis in mice (26). We demonstrated in this study that more IL-9 was secreted in *H. pylori-positive* gastritis patients, and IL-9 level was positively associated with mucosal inflammation. Among the co-stimulatory molecules, OX40 is reported to engage in IL-9 induction and promote the generation of Th9 cells (27, 28). We found that OX40 was highly up-regulated in the gastric mucosa of gastritis patients, consistent with the elevated level of IL-9 and increased number of MAIT cells. Further investigation indicated that OX40/OX40L signal induced the proliferation of IL-9 producing MAIT cells.

In this study, we investigated the potential role of IL-9 producing MAIT cells regulated by OX40/OX40L signal in *H. pylori*-induced gastritis. We identified a group of Th9-like MAIT cells in the gastric mucosa of gastritis patients with the ability to secrete IL-9, and elucidated that OX40/OX40L axis promoted proliferation and IL-9 production of MAIT cells. Collectively, our findings extended the understanding of the regulatory mechanism underlying the MAIT cells and provided possible interventions in clinical treatment of *H. pylori*-induced gastritis.

## Results

### Increased Number of IL-9-Producing MAIT Cells Was Found in the Gastric Mucosa of *H. pylori*-Positive Gastritis Patients

**Figure 1:**
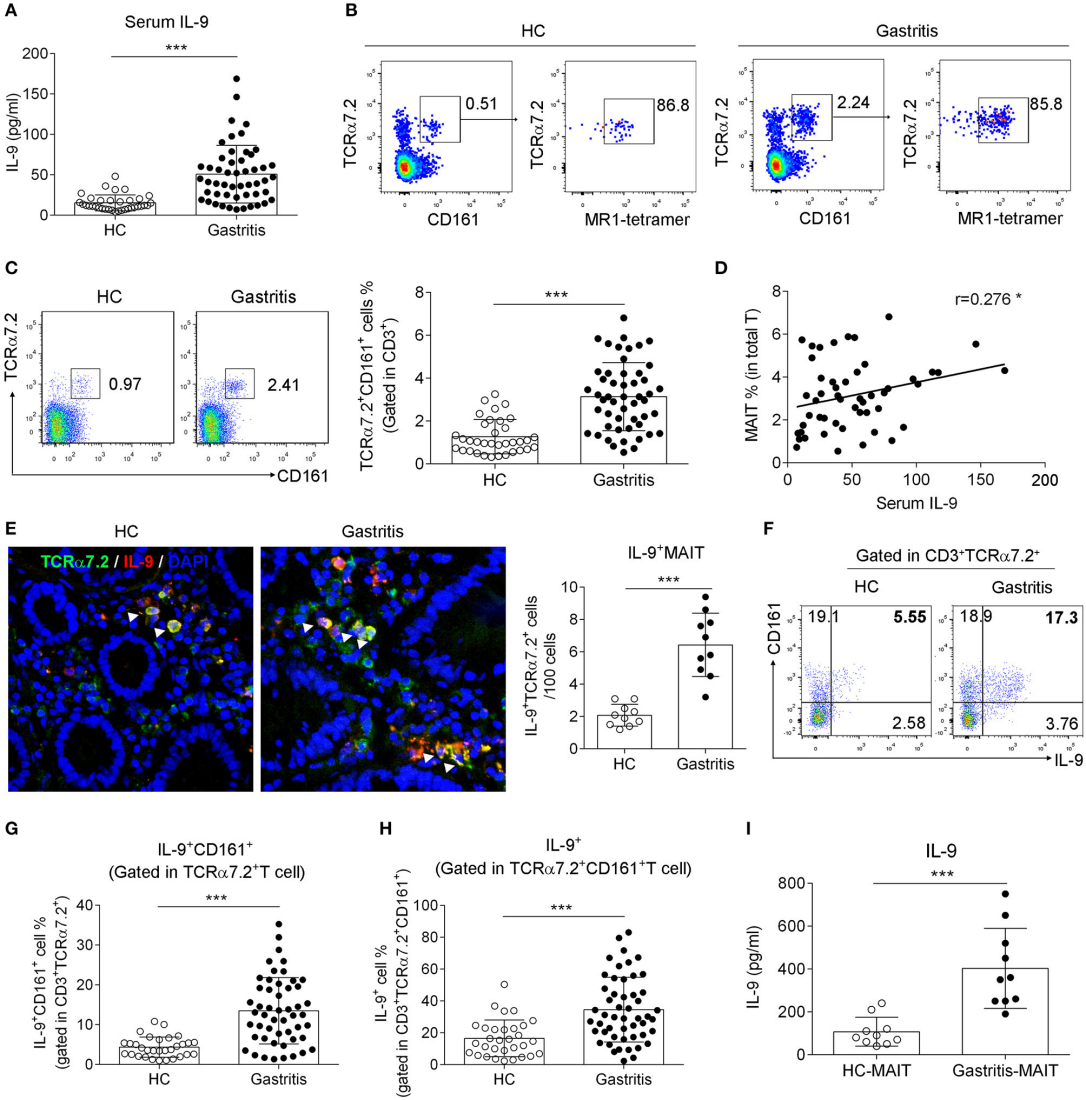
Increased IL-9-producing MAIT cells were found in the gastric mucosa of *H. pylori*-positive patients. Serum and the biopsy samples from gastric mucosa of *H. pylori*-positive patients (*n* = 51) and healthy controls (*n* = 35) were collected, respectively. **(A)** Serum IL-9 level was measured by ELISA. **(B)** The percentage of MR1-tetramer^+^ cells was determined in gastric lymphocytes gated on TCRa7.2^+^ CD161^+^ cells. **(C)** The percentage of MAIT cells in the gastric mucosa was determined by flow cytometry. **(D)** The correlation between the percentage of MAIT cells in the mucosa and serum IL-9 concentration was analyzed (*n* = 51). **(E)** Immunofluorescence was performed to evaluate the co-localization of MAIT cells (Green, indicated by TCRα7.2) with IL-9 (Red) (*n* = 10). Nucleus was stained with DAPI (Blue). Percentage of IL-9^+^ CD161^+^ cells (gated in TCRα7.2^+^ T cells) **(F,G)** and IL-9^+^ MAIT cells (gated in TCRα7.2^+^ CD161^+^ T cells) **(H)** were assessed by flow cytometry. **(I)** Sorted MAIT cells were stimulated by anti-CD3 and CD28 Abs for 12 h. IL-9 concentration in the culture supernatant of MAIT cells was tested by ELISA (*n* = 10). Data represented the mean ± S.D from at least three independent experiments. Unpaired Student's *t*-test was used to compare HC and gastritis groups. *P < 0.05; ***P < 0.001.

To evaluate the pattern of IL-9 in gastritis, we collected serum and biopsy samples from *H. pylori-*positive gastritis patients and healthy controls (The information of the patients and healthy controls was shown in Table 1), and tested IL-9 secretion. As expected, IL-9 level was elevated in the serum from *H. pylori* gastritis patients compared to healthy controls (Figure 1A). Meanwhile, increased percentage of MAIT cells (defined by both MR1-tetramer and TCRα7.2^+^ CD161^+^) was observed in biopsy samples of *H. pylori* gastritis patients (Figures 1B,C). To explore the relation of IL-9 with MAIT cells, we further analyzed the percentage of IL-9^+^ MAIT cells and the correlation between the percentage of MAIT cells and serum IL-9 level in *H. pylori* gastritis patients. Immunofluorescence assay showed that the co-localization of IL-9 with MAIT cells were increased in the gastric mucosa from *H. pylori* gastritis patients, and the percentage of MAIT cells in the mucosa was positively correlated with the concentration of serum IL-9 (Figures 1D,E). Furthermore, flow cytometry examination also demonstrated the increased percentage of IL-9^+^ CD161^+^ cells (gated in TCRα7.2^+^ T cells) (Figures 1F,G) and IL-9^+^ MAIT cells (gated in TCRα7.2^+^ CD161^+^ T cells) (Figure 1H) in the gastric mucosa of *H. pylori-*positive gastritis patients. More IL-9 secretion by mucosal MAIT cells was also confirmed with ELISA (Figure 1I). However, IL-9 expression was only slightly increased in conventional CD4^+^ and CD8^+^ T cells (Supplementary Figures 1A,B). Taken together, we found that MAIT cells of *H. pylori* gastritis patients secreted more IL-9 compared to healthy controls, indicating the necessity to further explore the role of IL-9 producing MAIT cells in *H. pylori* infection-induced gastritis.

**Table 1:** Characteristics of healthy donors and gastritis patients.

	**Healthy**	**Gastritis**	***P-*value**
Sample size (no.)	35	51	-
Age (years) (mean ± SD)	32.64 ± 14.36	39.52 ± 15.37	0.264
Sex (M/F)	12/23	25/26	0.192
**Indication for endoscopy (%)**			
Recurrent abdominal pain	NA	36 (70.6)	0.007^**^
Burning abdominal discomfort	NA	21 (41.2)	0.064
Acid reflux symptoms	NA	14 (27.5)	0.052
Dyspepsia	NA	23 (45.1)	<0.001^***^
Peptic ulcer	NA	27 (52.9)	<0.001^***^
Epigastic pain	NA	17 (33.3)	0.267
**Endoscopic finding (%)**			
Normal	35 (100)	0 (100)	<0.001^***^
Gastritis	0 (0)	51 (100)	<0.001^***^
^13^C-urea breath test positive (DOB>5) (%)	0 (0)	51 (100)	<0.001^***^
*H.pylori* infection (%)	0 (0)	51 (100)	<0.001^***^

*F, Female; M, male; DOB, Delta Over Baseline. The level of significance was evaluated by unpaired student t-test or Chi-square test. P-value < 0.05 was considered statistically significant*.

** *P < 0.001*.

*** *P < 0.0001*.

### OX40 Promoted IL-9 Production by Gastric MAIT Cells in *H. pylori*-Positive Gastritis Patients

**Figure 2:**
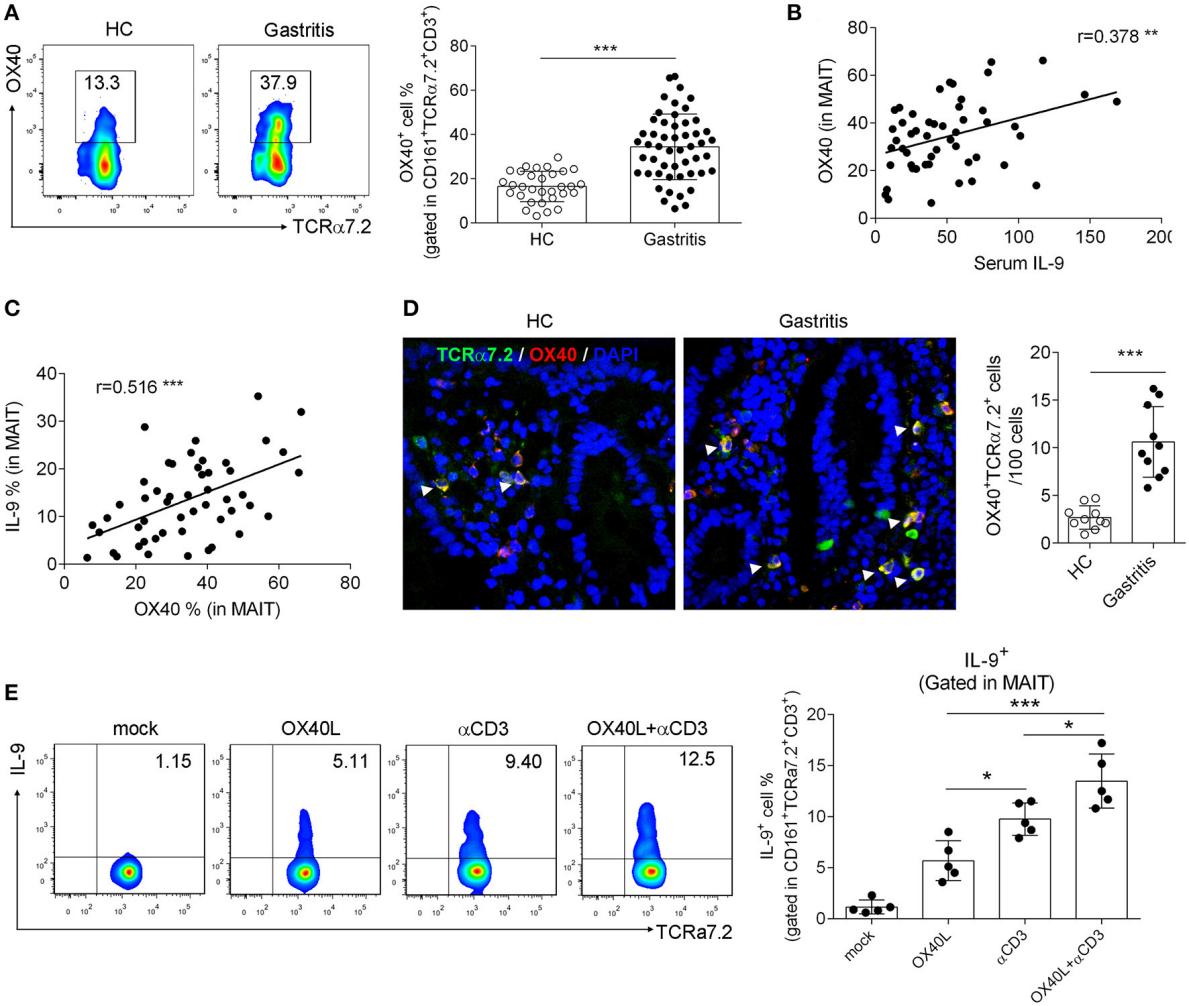
OX40 promoted IL-9 production by gastric mucosal MAIT cells in *H. pylori*-positive patients. Biopsies from gastric mucosa of *H. pylori*-positive patients (*n* = 51) and healthy controls (*n* = 35) were collected. **(A)** OX40 expression in MAIT cells was determined by flow cytometry. The correlations **(B)** between OX40 expression (in MAIT cells) and serum IL-9 level, and **(C)** between the percentage of OX40^+^ and IL-9^+^ MAIT cells were analyzed, respectively (*n* = 51). **(D)** Co-localization of OX40 and MAIT cells was assessed by immunofluorescence (*n* = 10). Antibodies against TCRα7.2 (Green), OX40 (Red), and DAPI (Blue) were used for staining. **(E)** MAIT cells were isolated from the gastric mucosa of *H. pylori*-positive patients (*n* = 5) and stimulated with 1 μg/ml CD3 activating Abs or 10 μg/ml recombinant OX40L protein. Three days later, IL-9 production by MAIT cells was determined. Data were from three independent experiments. Unpaired Student's *t*-test was used to compare the difference between HC and gastritis group. One way ANOVA was performed to compare multiple groups. *P < 0.05; **P < 0.01; ***P < 0.001.

We previously demonstrated that OX40 promoted MAIT cell activation in T2D patients (11). Besides, OX40 has been demonstrated to be associated with Th9 cell differentiation (28). To investigate whether OX40 promotes IL-9 production by MAIT cells in *H. pylori* gastritis, we next explored the change of IL-9 production after the intervention of OX40. We first analyzed the expression of OX40 in *H. pylori* gastritis. Consistent with the increased number of IL-9 producing MAIT cells, OX40 expression was also up-regulated in mucosal MAIT cells of *H. pylori* gastritis patients (Figure 2A). Further analysis indicated the positive correlations of the percentage of OX40^+^ MAIT cells with serum IL-9 level (Figure 2B) or the percentage of IL-9^+^MAIT cells (Figure 2C). Meanwhile, immunofluorescence staining also showed a high abundance of OX40 in mucosal MAIT cells of *H. pylori* gastritis patients (Figure 2D). We next isolated MAIT cells from the mucosa of *H. pylori* gastritis patients and treated MAIT cells with recombinant OX40L protein to activate OX40 expressed on MAIT cells and determined the production of IL-9. After treating MAIT cells with recombinant OX40L protein and CD3 activating antibody, the percentage of IL-9^+^ MAIT cells was increased (Figure 2E). However, there was no difference of OX40 expression in conventional CD4^+^ and CD8^+^ T cells between the HC vs. gastritis group (Supplementary Figures 1C,D). Based on the above results, we demonstrated that OX40 facilitated IL-9 production of mucosal MAIT cells in *H. pylori*-induced gastritis.

### OX40 Facilitated MAIT Cell Activation and Proliferation During *H. pylori* Infection

**Figure 3:**
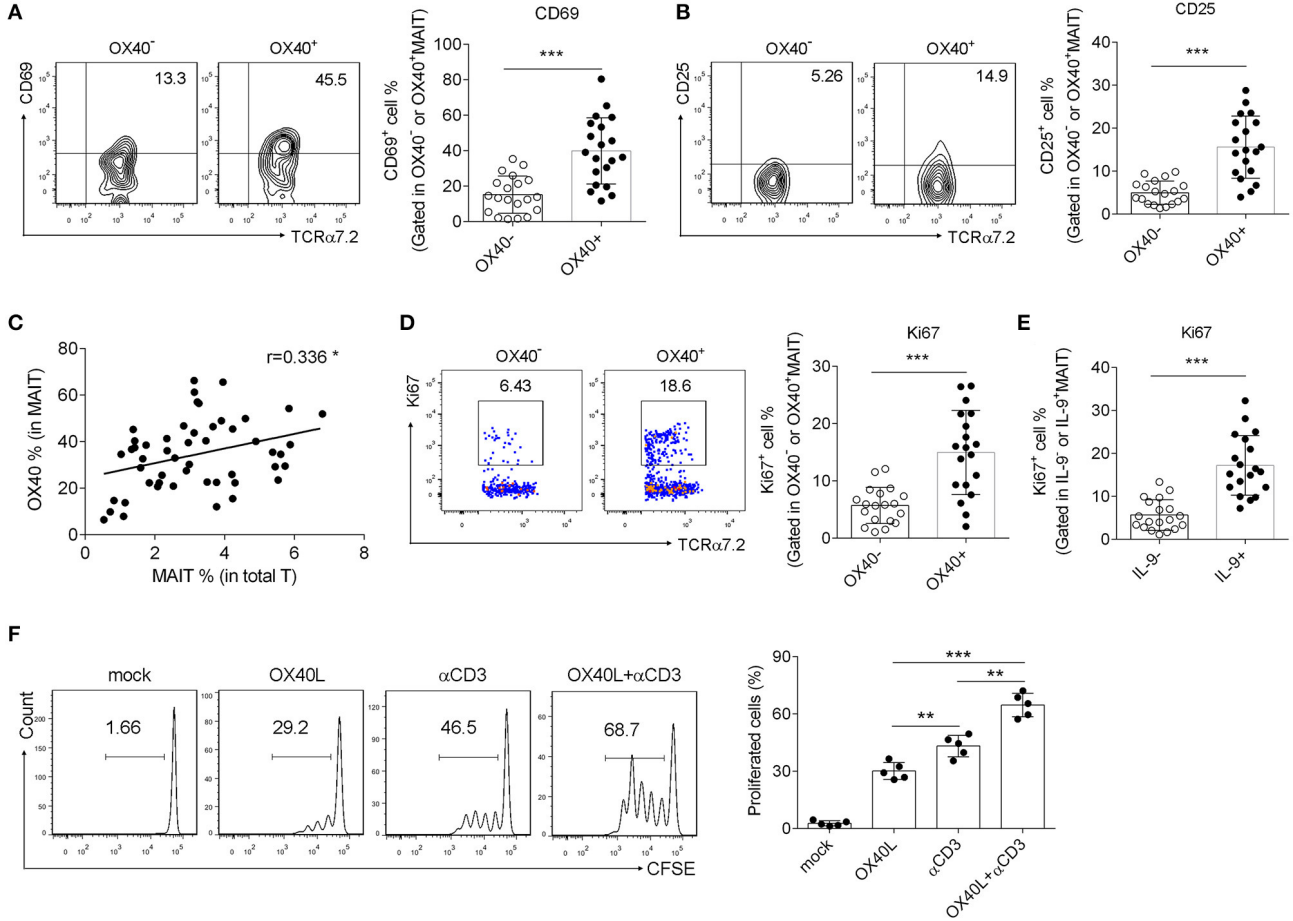
OX40 facilitated MAIT cell activation and proliferation in *H. pylori* infection. Gastric mucosa from *H. pylori*-positive patients (*n* = 20) was collected. **(A)** CD69 and **(B)** CD25 expression in OX40^−^ and OX40^+^ MAIT cells was determined by flow cytometry. **(C)** The correlation between OX40 expression in MAIT cells and the percentage of MAIT cells in total T cells was evaluated. Proliferation of **(D)** OX40^+^ and OX40^−^ MAIT cells, as well as **(E)** IL-9^+^ and IL-9^−^ MAIT cells were assessed, and reflected by the intensity of Ki67. **(F)** MAIT cells were isolated from the gastric mucosa of *H*. gastritis patients (*n* = 5) and stimulated with 1 μg/ml CD3 Abs or 10 μg/ml recombinant OX40L for 3 days. Proliferation of MAIT cells was determined, as represented by the fluorescence intensity of CFSE. Data represented the mean ±S.D from at least three independent experiments. Unpaired Student's *t*-test was used to compare the difference between HC and gastritis group. One way ANOVA was performed to compare multiple groups. **P < 0.01; ***P < 0.001.

Since OX40 promoted IL-9 production of MAIT cells, we then examined whether OX40 facilitated MAIT cell activation in *H. pylori*-induced gastritis, as what we found in T2D patients (11). As expected, OX40^+^ MAIT cells from *H. pylori* gastritis patients were found to express high levels of CD69 and CD25 (Figures 3A,B), which are T cell activating markers (29, 30). Meanwhile, a positive correlation was observed between the percentage of OX40^+^ T cells and MAIT cells (Figure 3C), indicating that OX40 may promote MAIT cell proliferation. To verify this possibility, we isolated MAIT cells (Supplementary Figure 2) and stained with Ki67, then analyzed the proliferation of OX40^+^ and OX40^−^MAIT cells by flow cytometry. Results showed that OX40^+^ MAIT cells had a higher ratio of proliferation compared to OX40^−^ MAIT cells (Figure 3D). Likewise, IL-9^+^ MAIT cells proliferated more than IL-9^−^ cells (Figure 3E), all together indicating that both OX40^+^ and IL-9-producing MAIT cells displayed higher proliferation abilities. We verified this conclusion by activating OX40 on MAIT cells with recombinant OX40L protein. Treatment of CD3 activating antibody and recombinant OX40L both facilitated MAIT cell proliferation, while combination treatment induced the highest level of MAIT cell proliferation (Figure 3F). Thus, we demonstrated that OX40 promoted MAIT cell activation and proliferation, and also facilitated IL-9 production by MAIT cells.

### OX40L Was Induced in Mucosal DCs and Promoted OX40-Mediated IL-9 Production and Proliferation of MAIT Cells During *H. pylori* Infection

**Figure 4:**
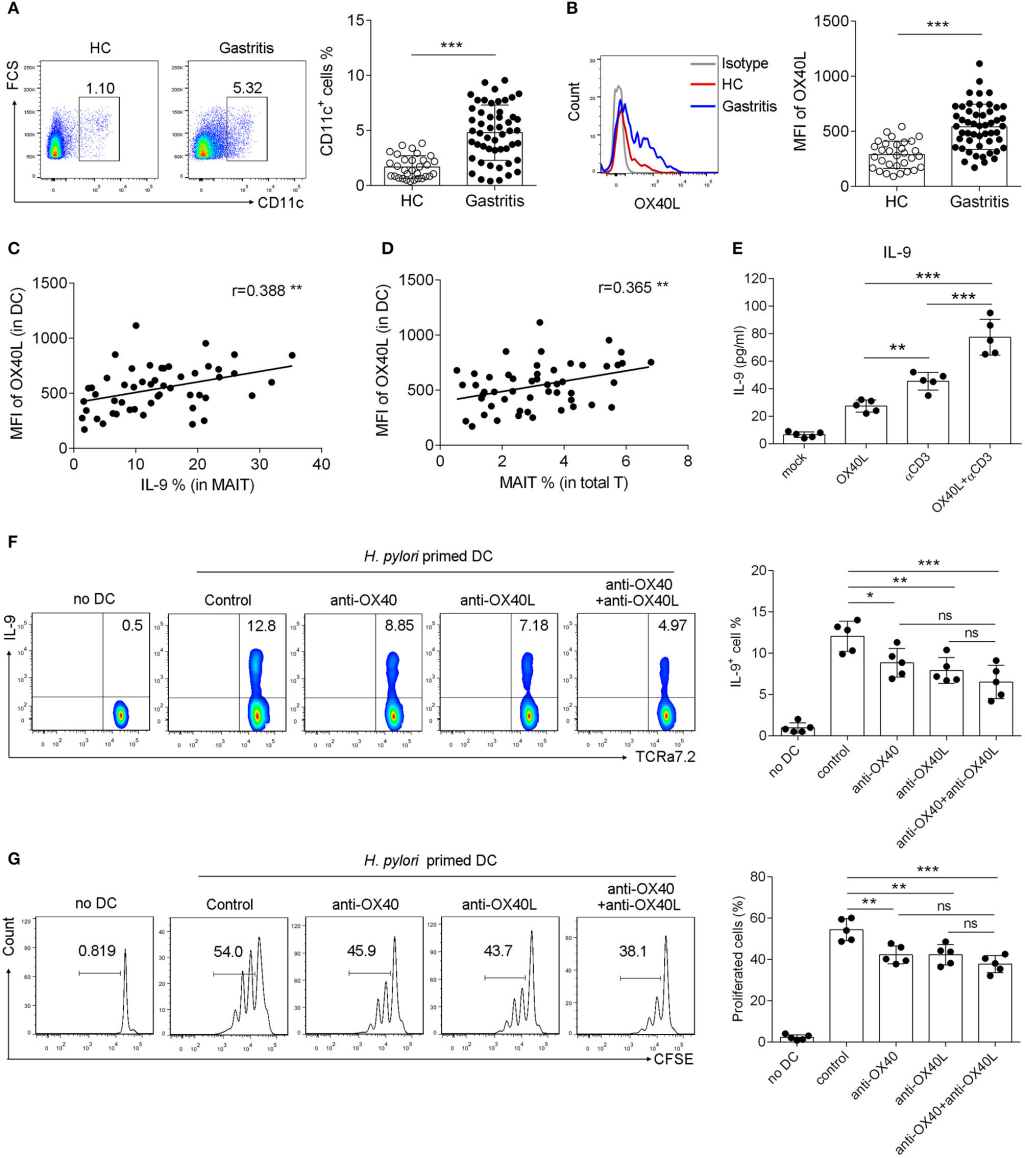
OX40L was induced in DCs and promoted OX40 mediated IL-9 production and proliferation of MAIT cells in *H. pylori* infection. DCs were isolated from the gastric mucosa of *H. pylori*-positive patients (*n* = 51) and healthy donors (*n* = 35). Flow cytometry was performed to detect the expression of **(A)** CD11c and **(B)** OX40L. The correlations of OX40L expression with **(C)** IL-9 production, and **(D)** MAIT cells numbers were analyzed, respectively (*n* = 51). **(E)** DCs were treated with 10 μg/ml recombinant OX40L protein to activate OX40, and IL-9 secretion was determined by ELISA 24 h later (*n* = 5). DCs were primed with *H. pylori* (MOI = 20) to be activated, and co-cultured with MAIT cells in the presence of blocking antibodies against OX40 (10 μg/ml) or OX40L (10 μg/ml). 5 days later, **(F)** IL-9 production and **(G)** proliferation of MAIT cells were assessed, respectively (*n* = 5). Data were from three independent experiments. Unpaired Student's *t*-test was used to compare the difference between HC and gastritis group. One way ANOVA was performed to compare multiple groups. *P < 0.05; **P < 0.01; ***P < 0.001.

To further explore the role of OX40/OX40L axis in the function of MAIT cells, we next investigated the expression and regulatory role of OX40L in *H. pylori*-induced gastritis patients. OX40 is mainly expressed on T cells, while its ligand OX40L is found predominately on myeloid cells, especially on DCs (31, 32). We first examined the expression of OX40L in mucosal DCs, which were defined as CD11c^+^ HLA-DR^+^ CD68^−^ CD16^−^ cells (Supplementary Figure 3). Increased number of DCs was observed in the mucosa from *H. pylori* gastritis patients, and OX40L expression on mucosal DCs was also up-regulated (Figures 4A,B). Simultaneously, positive correlations between OX40L expression in DCs and the percentage of IL-9^+^ MAIT cells or MAIT cells in the gastric mucosa were observed (Figures 4C,D). We also found that treatment of MAIT cells with recombinant OX40L protein induced IL-9 production (Figure 4E). Thus, we speculated that OX40L was also critical in IL-9 production of MAIT cells. To investigate this, we next established the co-culture system to evaluate the effect of OX40/OX40L signal on MAIT cell activation. DCs was pre-treated with *H. pylori* to load antigen, then co-cultured with MAIT cells in the presence of OX40 and OX40L blocking antibodies, followed by the measurement of MAIT cell proliferation and IL-9 production. Either OX40 or OX40L blockage inhibited MAIT cell proliferation and IL-9 production (Figures 4F,G), indicating the critical role of the OX40/OX40L signal in MAIT cell activation and IL-9 secretion in *H. pylori*-induced gastritis.

### The Percentage of IL-9 Producing MAIT Cells Was Positively Correlated With the Levels of Pro-inflammatory Cytokines and Mucus in *H. pylori*-Positive Gastritis Patients

**Figure 5:**
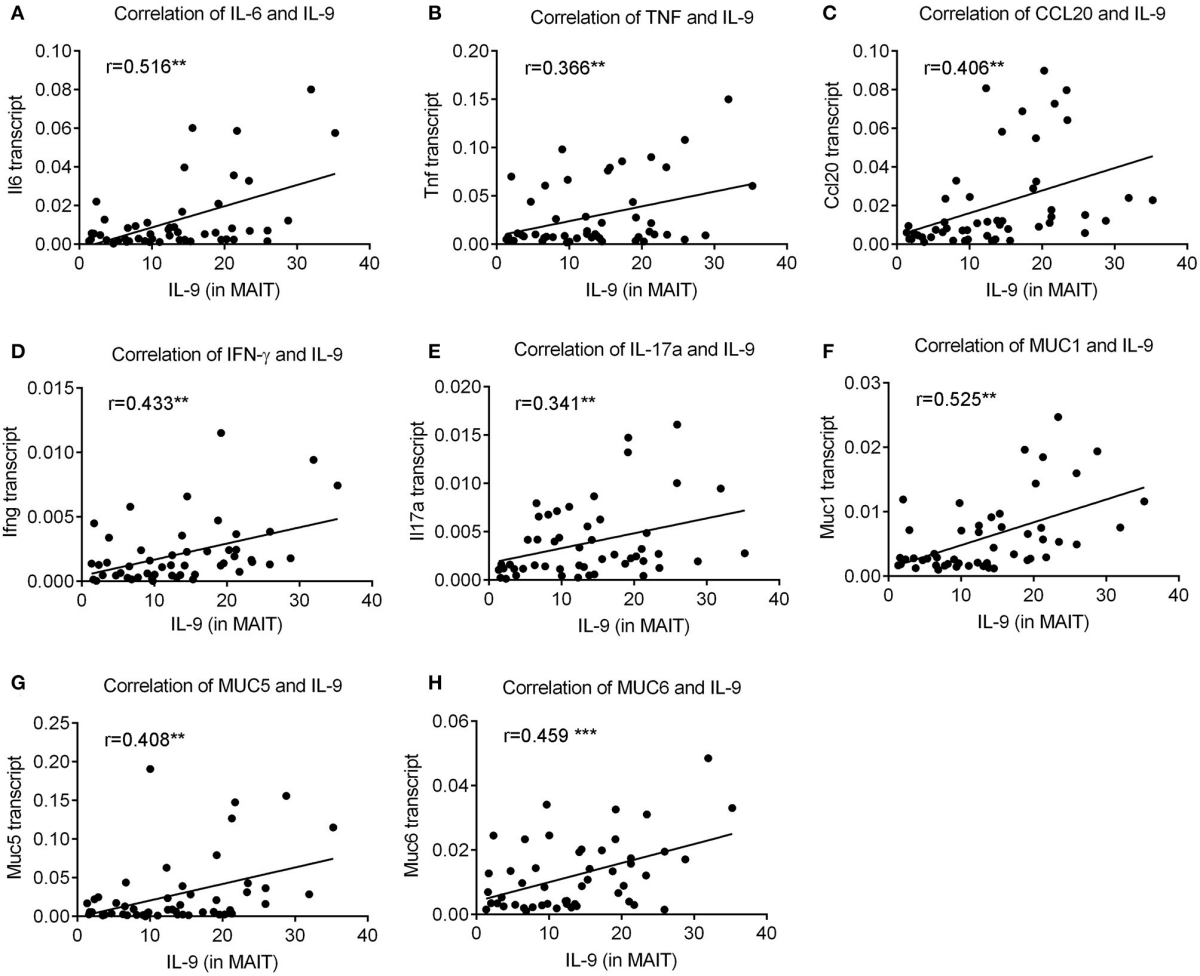
The percentage of IL-9 producing MAIT cells was positively correlated with expressions of pro-inflammatory cytokines and mucus in *H. pylori*-positive gastritis. The percentage of IL-9^+^ MAIT cells was determined by flow cytometry (*n* = 51). The correlations of IL-9^+^ MAIT cells with gene expressions of **(A)** IL-6, **(B)** TNF, **(C)** CCL20, **(D)** IFNγ, **(E)** IL-17a **(F)** MUC1, **(G)** MUC5, and **(H)** MUC6 were analyzed by spearman correlation analysis. **P < 0.01; ***P < 0.001.

Host infection by *H. pylori* initiates inflammatory responses to produce inflammatory effectors such as cytokines, chemokines, and mucus to eradicate the pathogens (33, 34). To further explore the relevance of IL-9-producing MAIT cells with *H. pylori*-mediated gastric mucosal inflammation, we analyzed the correlation between the percentage of IL-9^+^ MAIT cells and inflammatory indicators exhibited in *H. pylori* gastritis patients. We observed that the percentage of IL-9^+^ MAIT cells was positively correlated with inflammatory cytokines IL-6, TNF, IFNγ, and IL-17, chemokine CCL20, as well as mucus-related genes MUC1, MUC5, and MUC6 (Figure 5). Thus we hypothesized that IL-9^+^ MAIT cells might regulate the mucosal inflammation in *H. pylori*-mediated gastritis. Taken together, our research investigated the promoting effect of OX40-OX40L pathway on mucosal MAIT cell proliferation and IL-9 production during *H. pylori-*positive gastritis, providing potential strategies for clinical treatment of *H. pylori*-induced gastritis.

## Discussion

In this study, we investigated the potential role of OX40/OX40L axis in promoting IL-9 production and proliferation by mucosal MAIT cells in *H. pylori* gastritis patients. Our findings expanded the understanding of the underlying mechanisms involved in *H. pylori*-mediated gastritis, and provided potential intervening strategies for therapeutic purpose in *H. pylori* gastritis.

Except for Th9 cells, other types of cell such as innate lymphoid cells (ILCs) and NKT cells can also produce IL-9, but the underlying connections between IL-9 and its significance in physiological and disease conditions are still not clarified (24, 35). IL-9 is initially thought to be a T cell growth factor, mainly to promote T cell proliferation (36). Later, more evidences demonstrate that IL-9 can induce inflammatory responses and promote cytokine production, thus exerting regulatory functions in inflammation (25, 37, 38). It has been reported that transgenic expression of IL-9 in the lungs of mice resulted in extensive airway inflammation (28, 39), and IL-9 deficiency in Experimental autoimmune encephalomyelitis (EAE) mice reduces T cell infiltration and decreases IL-17 and IFN-γ production in CNS (40), which indicates that IL-9 plays critical roles in tissue inflammation. Our observations suggested the association of IL-9 with mucosal inflammation in *H. pylori*-positive gastritis. IL-9 level in the gastric mucosa of gastritis patients was positively correlated with mucosal inflammation, represented by up-regulated gene expression of pro-inflammatory cytokines and mucus-secreting related molecules. This research collectively suggests that IL-9 may serve as an inflammation amplifier to promote immune cell-mediated inflammatory responses, rather than only a growth factor to enhance T cell function.

IL-9 can be secreted by innate-like adaptive cells such as ILC cells and NKT cells. In this research, we demonstrated that MAIT cells can also produce IL-9, which expanded the range of IL-9 producing cells. After TCR ligation to riboflavin intermediates presented by MR1, MAIT cells are activated and expand rapidly to induce innate-like immune responses and exert effector functions by producing inflammatory cytokines and anti-microbial effector molecules (41, 42). Earlier studies have reported that MAIT cells in gastric mucosa can recognize *H. pylori*-infected macrophage, and produce IFN-γ and TNFα (19). Our findings revealed that mucosal MAIT cells secreted more IL-9 in *H. pylori* gastritis patients, and increased IL-9^+^ MAIT cells are positively correlated with mucosal inflammation. All together, this research implies that cytokines produced by mucosal MAIT cells are critical in *H. pylori* infection and gastritis, which needs to be determined by further studies.

Similar to the conventional T cells, except for the first signal transmitted by MR1-TCR, the co-stimulatory signal is required to fully activate MAIT cells (41, 43, 44). Co-stimulatory signals include co-stimulatory molecule pairs, Toll-like receptors, cytokines, etc. (1, 44). We previously demonstrated that OX40^+^ MAIT cells exhibited increased activation and memory phenotype in T2D patients (11). Meanwhile, it has been proved that OX40 can induce IL-9 production by Th9 cells (28), which both inspired us to explore the role of OX40 in mucosal resident MAIT cells of gastritis patients. OX40 (also called CD134) belongs to the TNFR superfamily, and delivers the co-stimulatory signal to mediate T cell activation, differentiation, and memory formation (45–47). When DCs were-pretreated with *H. pylori*, and then co-cultured with MAIT cells in the presence of blocking antibodies against OX40 and its ligand OX40L, proliferation and IL-9 production of MAIT cells decreased, suggesting that OX40/OX40L axis promoted proliferation and IL-9 production of MAIT cells. Combined with our findings in T2D patients that OX40 amplified activation-induced cell death (ACID) of MAIT cells in peripheral blood, we proposed OX40/OX40L axis as a crucial regulator of MAIT cells, by promoting MAIT cell activation and cytokines production. However, as OX40 may lead to excessive inflammation and hyperactivation of MAIT cells, which may lead to tissue damage and organ injuries (48, 49), the roles of OX40 in different diseases should be verified carefully through rigorous experiments both *in vitro* and *in vivo*.

MAIT cells have been a hotspot due to the potent abilities to regulate immune responses in inflammation and infectious diseases. Besides, the properties of MAIT cells, including their mucosal distribution and their intrinsic effector-memory phenotype, make them excellent candidates to harness in the development of vaccines (1). Our research investigated the function of MAIT cells in *H. pylori*-induced gastritis. We identified a group of IL-9 producing mucosal MAIT cells in *H. pylori* gastritis patients, and clarified the facilitating role of OX40/OX40L on proliferation and IL-9 production of MAIT cells. However, whether IL-9 producing MAIT cells promote mucosal inflammation was not investigated in this study, which is important to fully elucidate the function of MAIT cells in the gastric mucosa of *H. pylori* gastritis patients. In conclusion, our findings shed light on the function and potential regulatory mechanism of MAIT cells in gastritis, and hold implications for the clinical treatment of *H. pylori*-induced gastritis.

## Materials and Methods

### Ethics Statement

This study was approved by the Ethics Committee of Guangzhou Women and Children's Medical Center, Guangzhou Medical University (approval number 2017021709). Biopsy specimens from *H. pylori*-positive patients and healthy controls were collected from Guangzhou Women and Children's Medical Center (Guangzhou, China). Informed written consents were obtained from participants prior to commencement of the study.

### Subjects

This study enrolled 35 healthy donors and 51 gastritis patients who showed chronic symptoms of peptic disease, including dyspepsia, recurrent abdominal discomfort, and pain. Exclusive criteria included a history of acute onset of symptoms, acute or chronic vomiting, or the use of antibiotic, antacid, H2 blockers, proton-pump inhibitors, bismuth-containing compounds, or non-steroidal anti-inflammatory drugs within the preceding 4 weeks. Biopsy samples were obtained from the patients with indications for gastroscopy examination and were stained with Giemsa dye to observe the bacteria under a light microscopy. All of the 51 patients were diagnosed as *H. pylori*-positive gastritis with pathological changes of mucosa, whereas 35 healthy controls are with *H. pylori*-negative results and normal mucosa (See Table 1). Gastritis was diagnosed when the depth of damaged mucosal tissue was <5 mm, while the depth of damaged tissue was >5 mm in gastric ulcer patients (21).

### Preparation of Mucosal Mononuclear Cells

Mucosal mononuclear cells were isolated from the biopsy specimens of gastric mucosa of patients as we previously described (50). In brief, the biopsies were digested at 37°C for 45 min with shaking. The enzymatic digestion solution contained 1 ml of RPMI 1640 (Gibco), 10 μl of Collagenase D (100 μg/ml; Sigma-Aldrich, St. Louis, MO, USA) and 1 μl of DNase I (10 μg/ml; Thermo Fisher Scientific, Waltham, MA, USA). After the digestion, cells were collected through a 70 μm cell filter and centrifuged at 1,500 rpm. Collected cells were washed twice and re-suspended with basic RPMI 1640 medium for the next steps, including cell sorting, flow cytometry, immunofluorescence staining and co-culture assay (see below).

### MR1-Tetramers Staining

Human MR1-5-OP-RU labeled with BV421 or PE and MR1-tetramers were generated as described previously (2, 51). Briefly, refolded and purified empty carboxy-terminal cysteine-tagged-MR1 was loaded with a 136 molar excess of synthetic 5-OP-RU for 4 h at room temperature in the dark. For co-staining with MR1-tetramers, ~1 × 10^5^ cells were stained with MR1-5-OP-RU tetramer at 20 μg/ml for 40 min at room temperature in the dark. Cells were then washed and stained with anti-CD3, CD161, and TCRα7.2 for 30 min at 4°C. Cells were then washed once with 2 ml of PBS wash (2% fetal bovine serum in PBS) and resuspended in 150 ml of FACS fix (2% glucose and 1% paraformaldehyde in PBS) before acquisition of data on a BD LSR-Fortessa.

### Cell Sorting

Isolation of MAIT cells from mucosal mononuclear cells was performed by positive selection using the magnetic cell sorting system of BD Biosciences. TCRVα7.2^+^ cells CD161^+^ cells (MAIT cells) were purified by flow cytometry with anti-human TCRVα7.2 and CD161 antibodies (BD). The purity of sorted MAIT cells (defined as TCRVα7.2 and CD161 positive cells) was analyzed by flow cytometry, gated on CD3^+^ T cells (Supplementary Figure 1).

CD14^+^ cells were isolated from peripheral blood mononuclear cells (PBMCs) and purified by positive selection with human CD14 magnetic particles (BD) to generate dendritic cells (DCs), as we performed previously (52, 53). DCs were differentiated from CD14^+^ monocytes in the presence of 10 ng/μl granulocyte-macrophage colony-stimulating factor (GM-CSF) and 10 ng/μl IL-4 for 5 days.

### Co-culture Assay

*H. pylori* (MOI = 20) were incubated with mature DCs for 24 h. Then DCs were irradiated, extensively washed and used in co-culture with MAIT cells. Sorted MAIT cells were stained with 1 μM CFSE (Invitrogen, USA) and cultured in a 96-well flat-bottom plate. DCs and MAIT cells were co-cultured at a ratio of 1:5 (DCs, 2 × 10^3^ cells per well; MAIT cells, 1 × 10^4^ cells per well). We calculated the percentage of divided MAIT cells by gated on live TCRa7.2^+^ T cells.

To study the effect of OX40 on proliferation and IL-9 production by MAIT cells, recombinant human OX40 Ligand (10 μg/ml, R&D Systems), blocking antibodies of OX40 (10 μg/ml, Clone 977974, R&D Systems), and OX40L (10 μg/ml, Clone 159403, R&D Systems) were added into the co-culture system. Five days later, proliferation and IL-9 secretion of MAIT cells were determined by flow cytometry.

### Flow Cytometry Analysis

The procedure for cell staining in this study was described previously (53). For IL-9 staining, MAIT cells were restimulated for 12 h with 1 μg/ml anti-CD3 mAb (Clone UCHT1, BD), 1 μg/ml anti-CD28 mAb (Clone CD28.2, BD) and 3 μg/ml brefeldin A (eBioscience, CA, USA). IL-9 staining was performed with the intracellular fixation/permeabilization buffer set (eBioscience, CA, USA). Flow cytometric analysis was performed on FACS Canto II (BD, NJ, USA), and data were analyzed using FlowJo software (Tree Star). Involved anti-human antibodies were purchased from eBioscience, BD Biosciences or Biolegend (CA, USA): CD3 (Clone UCHT1, BD), CD161 (Clone HP-3G10, Biolegend), OX40 (Clone Ber-ACT35, Biolegend), OX40L (Clone ik-1, BD), CD11c (Clone B-ly6, BD), TCR Vα7.2 (Clone 3C10, Biolegend), and IL-9 (Clone Ber-ACT8, Biolegend).

### Immunofluorescence Staining and Confocal Microscopy

Paraffin-embedded samples were cut into 5-μm sections, and processed for immunohistochemistry as previously described (54). Briefly, tissues were fixed with 4% paraformaldehyde, followed by membrane permeabilization using 0.2% Triton-X-100. The coverslips were then incubated in 5% BSA, and were sequentially incubated with TCRa7.2 (Clone REA179, Miltenyi) and IL-9 (American Research Products, Product #E-AB-27215) or OX40 (Clone Ber-ACT35, Biolegend) Abs, followed by secondary Alexa Fluor^®^ 488 Ab (Thermo, Product # A-11034) or Alexa Fluor^®^ 594 Ab (Thermo, Product # A-11005) and DAPI (Thermo, Product # A-11034) staining before detection. Finally, the coverslips were observed under a ZEISS IMAGER A1 fluorescence microscope (CARL ZEISS) to capture fluorescence images.

### Real-Time PCR Analysis

Gastric mucosa was disrupted and homogenized by bead-milling as previously described (55). Total RNA was isolated using TRIzol reagent (Invitrogen) according to the manufacturer's recommendations. First Strand cDNA synthesis was performed using Synthesis Kit as described previously (52). For Real-time PCR, the expression of genes described in the literature (IL-6, TNF, CCL20, IFNγ, IL-17, MUC1, MUC5, MUC6, and β-actin) were assessed by PCR amplification with Bio-Rad CFX96 real-time detection system using SYBR Green Master Mix kit (Invitrogen). The level of target gene mRNA relative to β-actin was calculated using the following formula: Relative mRNA expression = 2^CTvalue(β−*actin*−*targetgene*)^.

### ELISA

Serum of gastritis patients and healthy controls enrolled in this study was collected and IL-9 concentration was determined by ELISA with Human IL-9 ELISA kit (Fitzgerald, Product # 55R-1973) according to the manufacturer's instructions. To determine the amount of IL-9 release, MAIT cells were isolated and restimulated with 1 μg/ml anti-CD3 mAb, 1 μg/ml anti-CD28 mAb, and 3 μg/ml brefeldin A for 12 h, and then the culture supernatant was collected for ELISA assay.

### Statistical Analysis

Statistical analysis was performed using GraphPad Prism 5.0 (GraphPad Software, San Diego, CA). Statistical significance was determined by Kruskal-Wallis, Chi-square test or Mann-Whitney non-parametric tests, and with analysis of one way analysis of variance (ANOVA) or unpaired Student's *t*-tests. The data are shown as the mean ± SD unless otherwise stated. *P*-value < 0.05 was considered significant.

## Data Availability Statement

The raw data supporting the conclusions of this article will be made available by the authors, without undue reservation.

## Ethics Statement

The studies involving human participants were reviewed and approved by the Ethics Committee of Guangzhou Women and Children's Medical Center, Guangzhou Medical University (approval number 2017021709). The patients/participants provided their written informed consent to participate in this study.

## Author Contributions

SM and YW wrote the manuscript. YW designed experiments. SM, MZ, ZL, CL, JH, XN, SD, and YW performed experiments and analyzed data. LG, PC, SZ, and GZ provided scientific expertise. YW and SG supervised the project. All authors contributed to the article and approved the submitted version.

## Conflict of Interest

The authors declare that the research was conducted in the absence of any commercial or financial relationships that could be construed as a potential conflict of interest.
