# Supplementary material for: OX40L/OX40 Signal Promotes IL-9 Production by Mucosal MAIT Cells During Helicobacter pylori Infection
Source: Front Immunol. 2021 Mar 11;12:626017. doi: 10.3389/fimmu.2021.626017 (PMC7990886; doi:10.3389/fimmu.2021.626017)
Supplement: Supplementary file 1 [file Data_Sheet_1.PDF]

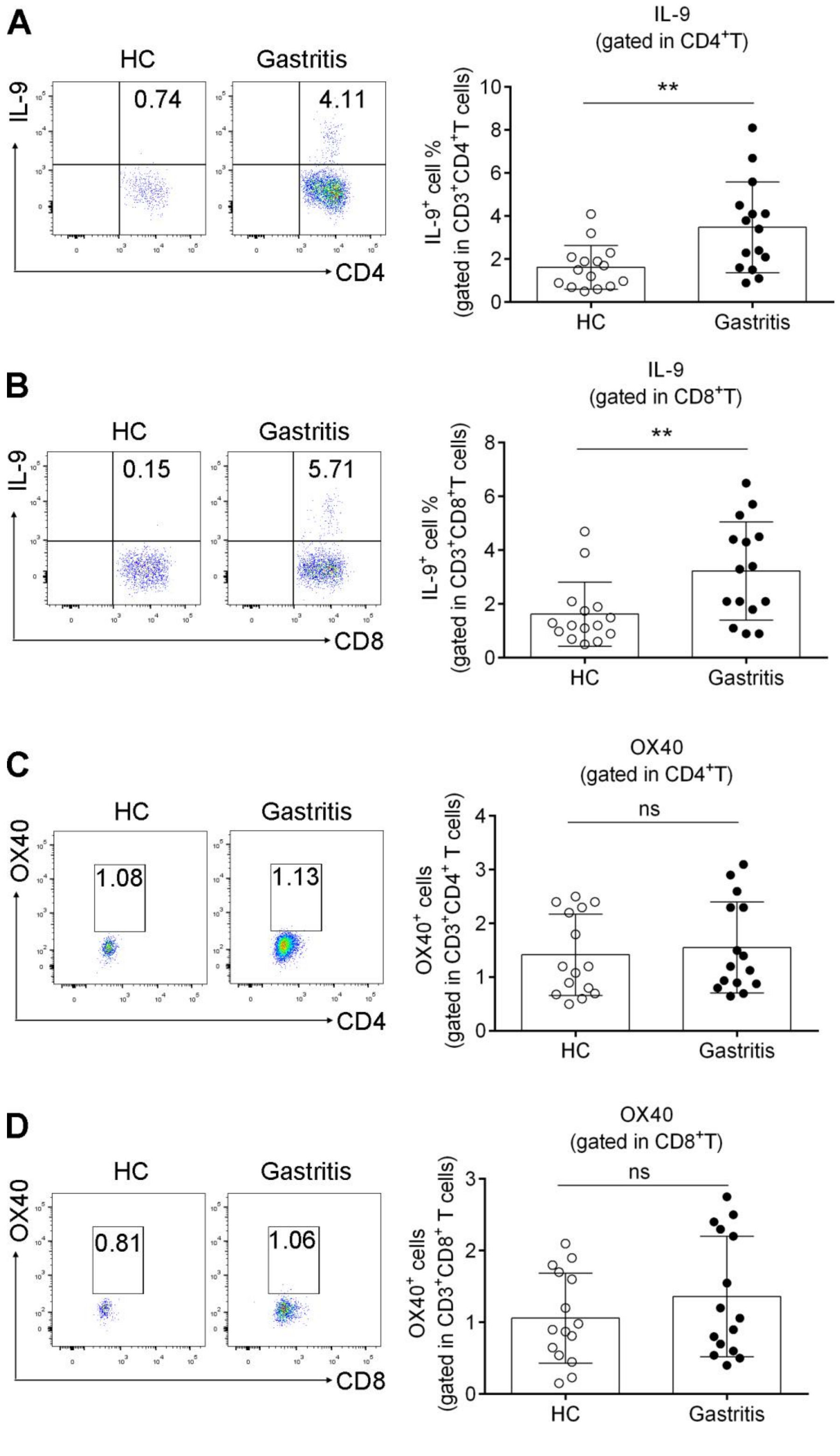

Supplementary Figure 1. The production of IL-9 and expression of OX40 in conventional $CD4^+$ and $CD8^+$ cells. **(A, B)** Gastric cells were stimulated by PMA and ionomycin for 6 hours. Intracellular IL-9 were detected in conventional $CD4^+$ and $CD8^+$T cells by flow cytometry gated

on $CD3^+$ $TCRa7.2^-$ cells. **(C, D)** The expression of OX40 was analyzed in $CD4^+$ and $CD8^+$ T cells by flow cytometry. Unpaired Student's t-test was used to compare HC and gastritis groups. ns, $p>0.05$. **, $p<0.01$.

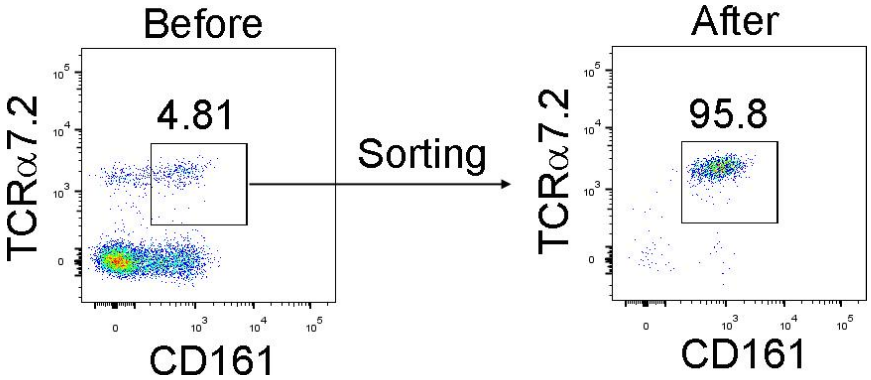

Supplementary Figure 2. The purify of MAIT cells. The expression of TCRa7.2 and CD161 in gastric cells before and after MAIT cell sorting was determined by flow cytometry.

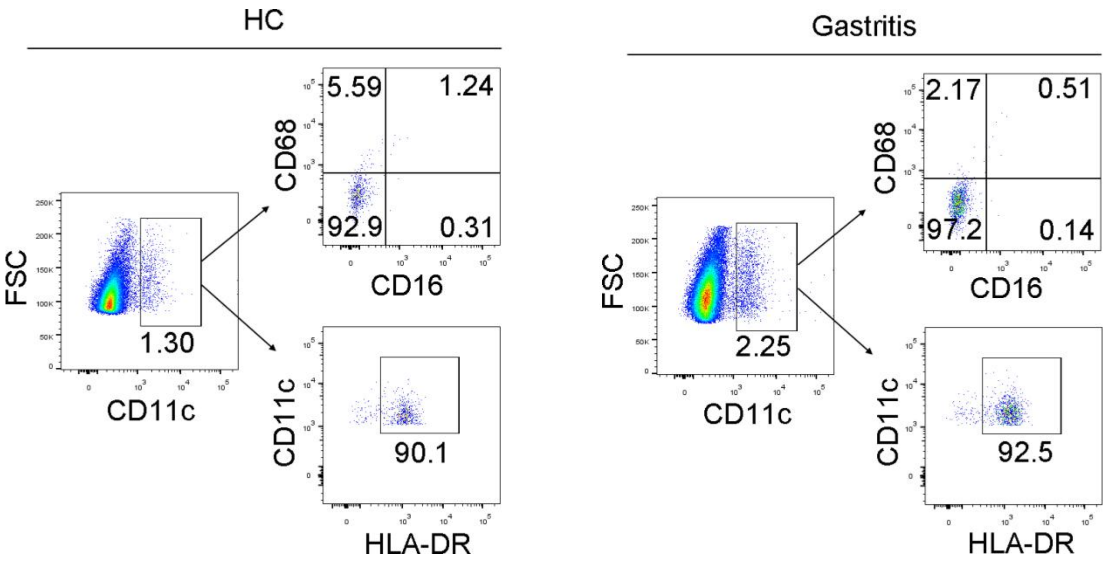

Supplementary Figure 3. The phenotype of CD11c^{+} DCs in gastritis patients and Healthy controls. The expression of HLA-DR, macrophage marker CD68 and neutrophil marker CD16 were determined by flow cytometry.
